# Smoking-Related DNA Alkylation Events Are Mapped at Single-Nucleotide Resolution

**DOI:** 10.1021/acscentsci.3c00246

**Published:** 2023-03-07

**Authors:** Alvin T. Huang, Weixin Tang

**Affiliations:** † Department of Chemistry, The University of Chicago, Chicago, Illinois 60637, United States; ‡ Institute for Biophysical Dynamics, The University of Chicago, Chicago, Illinois 60637, United States

**Figure 1:**
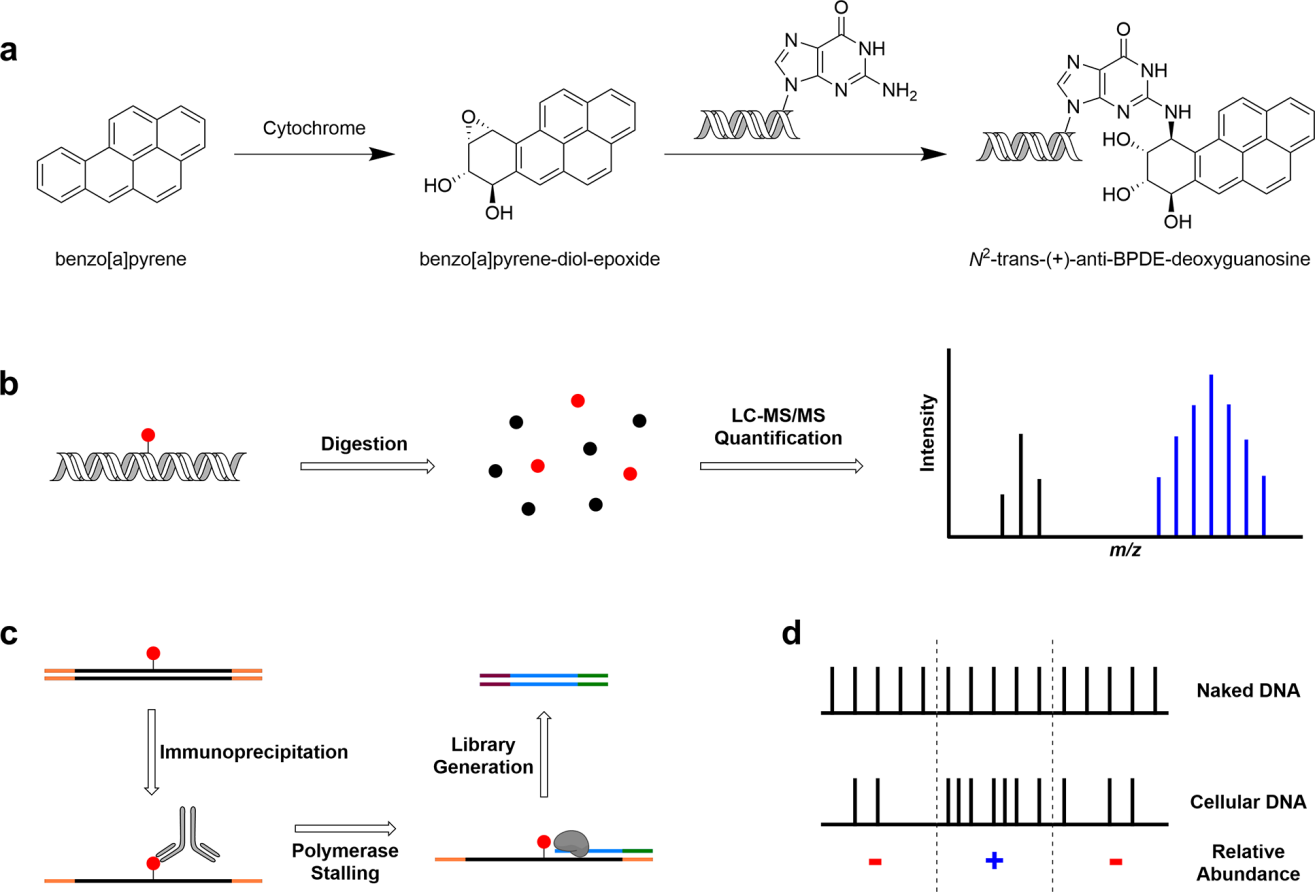
(a) BaP is metabolized by cytochrome enzymes to form the highly reactive BaP-diol-epoxide. A nucleophilic attack by the deoxyguanosine amine opens the epoxide ring forming *N*^2^-trans-(+)-anti-BPDE-deoxyguanosine in DNA. (b) To quantify the amount of adduct, genomic DNA is extracted, digested, and analyzed by mass spectrometry. (c) To analyze the adduct distribution, immunoprecipitation is performed on fragmented genomic DNA to separate the modified DNA. A DNA polymerase stalling assay is then performed to create shortened fragments that end immediately in front of the adduct. These short amplicons are barcoded, sequenced, and aligned to the genome to locate modified sites. (d) Sequencing data from naked and cellular DNA are overlaid, and the difference in abundance of adduct is noted. Regions modified to lower/higher levels in cellular DNA compared to naked DNA are marked with a red “–” and a blue “+”, respectively.

E xposure to environmental stressors can induce DNA damage, resulting in alterations to the genetic code. The accumulation of certain mutations, particularly in genes relevant to cell growth and division, can promote the development of various diseases including cancer.^1^ Some of the most well-studied carcinogens originate from cigarettes.^2^ One such chemical is benzo[a]pyrene (BaP), a polycyclic aromatic compound that is metabolized by cytochrome enzymes to form the incredibly reactive BaP-diol-epoxide (BPDE).^3,4^ BPDE irreversibly reacts with guanine to form a variety of ring-opened products, the most common being *N*^2^-trans-(+)-anti-BPDE-deoxyguanosine (*N*^2^-BPDE-dG) (Figure 1a).^5^ The adduct can be tracelessly removed through nucleotide excision repair, but those evading repair often lead to C:G to A:T mutations.^6^ Although the mechanism of damage is well understood, the exact locations of *N*^2^-BPDE-dG have not been mapped in a cellular context. In this issue of *ACS Central Science*, Sturla and co-workers report the first single-nucleotide resolution map of *N*^2^-BPDE-dG in the human genome which they use to elucidate the relationship between adduct formation and mutational signatures, especially those found in smoking-related lung cancers.^7^

The authors devised separate workflows to quantify the prevalence of *N*^2^-BPDE-dG and characterize its distribution. Quantification was done using liquid chromatography tandem mass spectrometry on cellular and naked genomic DNA of human bronchial epithelial cells (BEAS-2B) exposed to BPDE (Figure 1b). The *N*^2^-BPDE-dG adduct was identified in both samples, with naked DNA showing a higher abundance. Overall adduct levels increased with higher concentrations of BPDE. The behavioral discrepancy between naked and cellular DNA presumably involves cellular machinery such as histone, transcription, and repair proteins.

To reveal locations of *N*^2^-BPDE-dG, the authors developed BPDE-dG-Damage-seq (Figure 1c). Briefly, genomic DNA is fragmented followed by immunoprecipitation. The modified DNA is subsequently replicated with DNA polymerase that stalls at the location of the adduct, leading to shorter fragments that end immediately before *N*^2^-BPDE-dG. These fragments are then barcoded, sequenced, and aligned to the human genome to determine the location of damage. The high frequency of G at the called damage site across various BPDE concentrations, along with the lack of nucleotide enrichment at adjacent sites, supports the fidelity of BPDE-dG-Damage-seq.

In line with the mass spectrometry data, the level of damage in cellular DNA was almost always lower than that of naked DNA. The impact of cellular processes on damage accumulation was inferred from the relative abundance of *N*^2^-BPDE-dG in cellular and naked DNA (Figure 1d). Overlaying with a chromatin accessibility map revealed that open regions had fewer adducts compared to closed regions. This observation was largely consistent with the methylation state of the DNA. On the sequence level, highly transcribed genes carried less damage; the transcribed strand had less damage compared to the nontranscribed strand. These observations point to differences in how DNA is repaired: open chromatin is more accessible to the repair machinery compared to closed chromatin; the template strand may be preferentially repaired by transcription-coupled nucleotide excision repair.^8^

With this information in hand, the authors compared their genomic map with mutations identified in smoking-related lung cancers. The relative frequencies of *N*^2^-BPDE-dG-bearing trinucleotide sequences observed by the authors were remarkably similar to those of mutational signatures found in cancer patients.^2^*N*^2^-BPDE-dG was detected consistently in lung cancer hot-spot genes, including the chromatin modifying gene SMARCA4 and the tumor suppressor genes CDKN2A, KEAP1, and TP53. In particular, BPDE damage found in CDKN2A matches the most frequently mutated site of this gene in lung adenocarcinoma, which is also one of the top ranked genome-wide mutations observed in lung carcinomas. Almost all lung-carcinoma patients with mutations at this site are smokers, indicating a strong correlation between BPDE-mediated formation of DNA lesions and the progression of lung cancer.

Collectively, Sturla and co-workers construct a genome-wide map for *N*^2^-BPDE-dG that links carcinogen-DNA adducts to mutational signatures seen in medical cases. The study also provides a general method to genetically map DNA alkylation events and predict cancer mutations. A key insight of the study is that genomic features shape the formation and retention of alkylation products. DNase data from BEAS-2B cells, instead of a generic DNase map of the human genome as used by the present study, would enable a more specific analysis.

The fact that open DNA carries fewer adducts raises an intriguing paradox: unlike nucleosome-packed DNA, open DNA is less protected from damage, but is also more actively repaired. Results of the current study suggest that it is the repair process, rather than reactivity, that dominates the accumulation of alkylation products in the genome. It would be interesting to determine if such a trend persists with shorter treatment time or in different cell types. Many of the observed phenomena can be mechanistically dissected through time-resolved profiling and by comparing damage profiles in cells of active and disabled repair machinery. Ultimately, these studies will provide important insights into the relationship between DNA alkylation, damage accumulation, and cancer progression.
